# Supplementary material for: Comparative multiomics analysis of cell physiological state after culture in a basket bioreactor
Source: Sci Rep. 2022 Nov 23;12:20161. doi: 10.1038/s41598-022-24687-4 (PMC9686226; doi:10.1038/s41598-022-24687-4)

23666045.1:79418845:79419246:-@NW_023666045.1:79418206:79419246:-@NW_023666045.1:79418206:7941

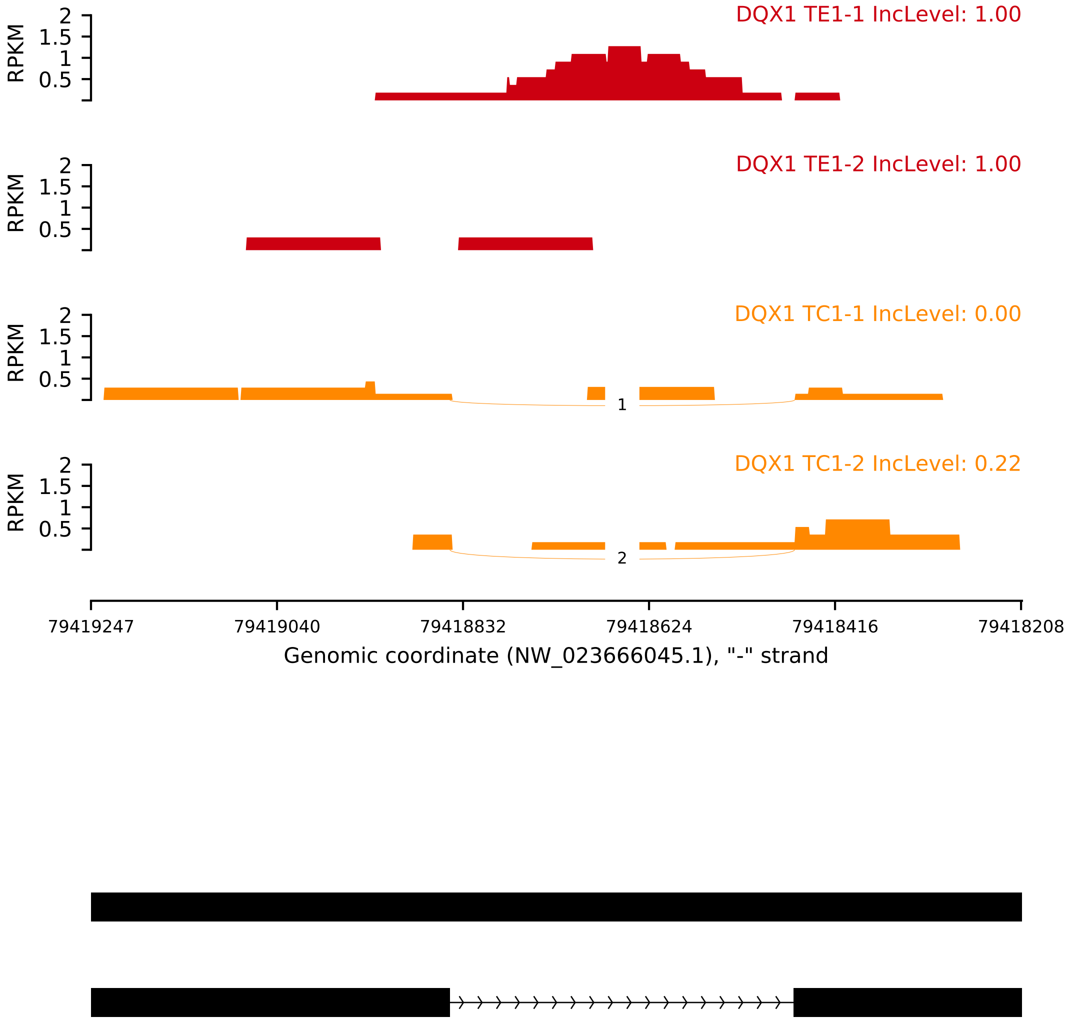

Supplement: Supplementary file 1 — Supplementary Information 1. [file 41598_2022_24687_MOESM1_ESM.zip › raw data/Transcriptomic raw data/7.AS/2.dasplot/TE1vsTC1/RI/11_DQX1_NW_023666045.1_79418845_79419246_-@NW_023666045.1_79418206_79419246_-@NW_023666045.1_79418206_79418461_-.pdf]
